# On Modern Progress in Ophthalmic Medicine and Surgery

**Published:** 1894-02-10

**Authors:** Robert Brudenell Carter

**Affiliations:** Consulting Ophthalmic Surgeon to St. George's Hospital


### ON MODERN PROGRESS IN OPHTHALMIC MEDICINE AND SURGERY.

By ROBERT BRUDENELL CARTER, F.R.C.S., Consulting Ophthalmic Surgeon to St. George's Hospital.

DURING the progress of the changes described in my last paper, and brought about by the steady increase of pressure upon the ocular nerves and blood-vessels, the relative positions of structures within the eye are likely to undergo considerable alteration. Partly because the secretion of fluid is largely effected by the ciliary processes, and partly on account of the continued, if impeded, entrance of blood through the central artery of the retina, the tension increases more rapidly behind the lens than in front of it; and hence the lens and iris are pushed forward, and made to encroach upon the anterior chamber, while the pupil often assumes an elliptical shape with its major axis horizontal, and seems to be mechanically dilated in consequence of the lens being pushed through it from behind. At the same time, the nutrition of the corneal epithelium is often impaired, so that its transparency is diminished, and the corneal surface looks a little cloudy or steamy, as if it had been breathed upon. Such a condition materially increases any pre-existing impairment of vision, and practically excludes the use of the ophthalmoscope.

The several symptoms above-mentioned may be developed in varying order, with different rates of rapidity in different cases, and sometimes even so slowly that their true character is in danger of being overlooked. If the increase of ocular tension be very slow, it may be quite painless, and sight may fade away by almost imperceptible degrees. Such a condition, before its glaucomatous character was understood, was described as "atrophy with excavation of the optic nerve." There may be very little vascular disturbance, the course of the blood having time to adjust itself to the altered conditions. At the other end of the scale we find the form of glaucoma described by Von Graefe as "fulminating," in which the increase of tension is so rapid as to produce agonising pain by stretching the ocular tunics, in which blindness is complete from the onset of the attack, and in which it becomes permanent unless an effectual iridectomy be accomplished within a period of three or four hours. The most common forms are intermediate between the foregoing, and the tension usually mounts up by successive small and almost sudden increments. After a period of increasing presbyopia, often accompanied by an appearance of rainbow rings around a candle flame, the patient will complain of a cloud before one eye, or of a state of darkness of it, which may amount to blindness for a time. There will probably be pain, and also surface congestion, or as it will often be called, "inflammation." After a while, from a few minutes to as many hours, the vision may be restored, the pain may subside, and the surface congestion may disappear. The patient will fancy himself well again; but careful examination will show that vision has not quite returned to the normal standard, that the pupil is sluggish, and that the eye is a little hard; while the ophthalmoscope may reveal pulsation of vessels on the nerve surface, or even the commencement of excavation, shown by some bending of the vessels at the edge of the opening through which they pass. In a few weeks or months the attack will probably recur, it may be with greater or with less severity; and, when it has again passed away, the sight will be a little worse than before. If such warnings are neglected they are likely to be followed by a final attack, from which no recovery may be possible.

For many years after the discovery of iridectomy, ophthalmic surgeons were exposed to the pain of seeing cases such as those above described, but of seeing them only at a time when all prospect of improvement had passed away. The pain was mistaken for "rheumatism" or for "gout," the congestion for "inflammation," and the patient was withheld from proper treatment while the supposed constitutional malady was being combatted by alkalies or by colchicum, or while the supposed inflammation was being aggravated by belladonna. Errors of this kind, even now, are unfortunately not wholly impossible; and the responsibility for them is largely due to certain teachers who have laid undue stress upon the diathetic character of many eye affections. The diathesis is generally doubtful; but the pupil, unskilled in ocular diagnosis, jumps at the diathesis and neglects the eye. In order that such mistakes, as far as glaucoma is concerned, should not occur, the chief thing necessary is that every medical practitioner should be adequately skilled in the simple art of estimating ocular tension, and that he should estimate it, almost as a matter of routine, in every case of ocular trouble or disease about which he may be consulted.

The estimation of tension is accomplished by the combined use of the two forefingers. The patient should sit with drooping lids, looking down towards the ground, while the surgeon gently places the tip of one forefinger upon the upper lid, as far back towards or under the margin of the orbit as it will go, and with this steadies or fixes the eyeball. With the tip of the second forefinger, placed by the side of the first, he gently and carefully presses the eyeball, and feels the amount of resistance by which the pressure is opposed. A healthy eye will easily dimple somewhat under the finger; a completely glaucomatous eye will be as hard as a stone, and, indeed, is very suggestive of the sensation which would be given by a child's marble under the lid. Between these there are many gradations, and, for the purpose of casetaking, Sir William Bowman described four, and indicated them by using T for the word tension, with a plus or minus sign prefixed, and a note of interrogation or a numeral following to denote the degree. For normal tension he had "Tn.," and for degrees of increase +T?, +T1, +T2, and +T3. The method is convenient, if nothing more. Estimation of the degree is facilitated by comparing the affected eye with its fellow, although, in doing this, it must not be forgotten that both eyes often participate in the glaucomatous condition, and that one may be only a little more advanced than the other. If a standard be required, the observer may take either his own eye, or that of any other healthy person who is at hand; and the existence of morbid tension will hardly escape notice, if once the desirability of feeling for it has been remembered. When morbid tension is present, the case admits of no temporising, and usually of but little delay. For a patient who has seen rainbows, who has had more or less complete obscuration of vision, and whose eye is hard, a short period of time may often be gained by the sedulous application of eserine. But such a period will at best be short, and the only security for the preservation of sight must be sought in the prompt performance of iridectomy or of sclerotomy. The longer either operation is delayed the less beneficial, generally speaking, will be its effects.

